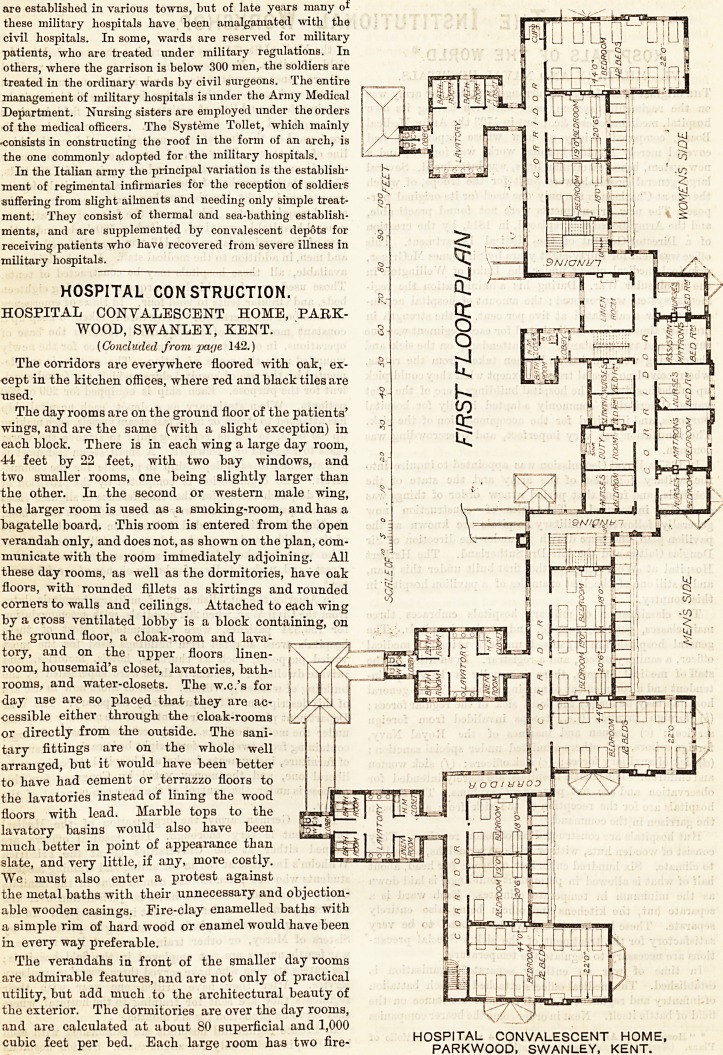# Hospital Convalescent Home, Parkwood, Swanley, Kent

**Published:** 1893-12-09

**Authors:** 


					HOSPITAL CONSTRUCTION.
HOSPITAL CONVALESCENT HOME, PARK-
WOOD, SWANLEY, KENT.
(Concluded from page 142.)
The corridors are everywhere floored with oak, ex-
cept in the kitchen offices, where red and black tiles are
used.
The day rooms are on the ground floor of the patients'
wings, and are the same (with a slight exception) in
each block. There is in each wing a large day room,
44 feet by 22 feet, with two bay windows, and
two smaller rooms, one being slightly larger than
the other. In the second or western male wing,
the larger room is used as a smoking-room, and has a
bagatelle board. This room is entered from the open
verandah only, and does not, as shown on the plan, com-
municate with the room immediately adjoining. All
these day rooms, as well as the dormitories, have oak
floors, with rounded fillets as skirtings and rounded
corners to walls and ceilings. Attached to each wing
by a cross ventilated lobby is a block containing, on
the ground floor, a cloak-room and lava-
tory, and on the upper floors linen-
room, housemaid's closet, lavatories, bath-
rooms, and water-closets. The w.c.'s for
day use are so placed that they are ac-
cessible either through the cloak-rooms
or directly from the outside. The sani-
tary fittings are on the whole well
arranged, but it would have been better
to have had cement or terrazzo floors to
the lavatories instead of lining the wood
floors with lead. Marble tops to the
lavatory basins would also have been
much better in point of appearance than
slate, and very little, if any, more costly.
We must also enter a protest against
the metal baths with their unnecessary and objection-
able wooden casings. Fire-clay enamelled baths with
a simple rim of hard wood or enamel would have been
in every way preferable.
The verandahs in front of the smaller day rooms
are admirable features, and are not only of practical
utility, but add much to the architectural beauty of
the exterior. The dormitories are over the day rooms,
and are calculated at about 80 superficial and 1,000
cubic feet per bed. Each large room has two fire-
are established in various towns, but of late years many of
these military hospitals have been amalgamated with the
civil hospitals. In some, wards are reserved for military
patients, who are treated under military regulations. In
others, where the garrison is below 300 men, the soldiers are
treated in the ordinary wards by civil surgeons. The entire
management of military hospitals is under the Army Medical
Department. Nursing sisters are employed under the orders
of the medical officers. The Systeme Toilet, which mainly
?consists in constructing the roof in the form of an arch, is
the one commonly adopted for the military hospitals.
In the Italian army the principal variation is the establish-
ment of regimental infirmaries for the reception of soldiers
suffering from slight ailments and needing only simple treat-
ment. They consist of thermal and sea-bathing establish-
ments, and are supplemented by convalescent depots for
receiving patients who have recovered from severe illness in
military hospitals.
HOSPITAL CONSTRUCTION.
HOSPITAL CONVALESCENT HOME, PARK-
WOOD, SWANLEY, KENT.
(Concluded from page 142.)
The corridors are everywhere floored with oak, ex-
cept in the kitchen offices, where red and black tiles are
-used.
The day rooms are on the ground floor of the patients'
wings, and are the same (with a slight exception) in
each block. There is in each wing a large day room,
44 feet by 22 feet, with two bay windows, and
two smaller rooms, one being slightly larger than
the other. In the second or western male wing,
the larger room is used as a smoking-room, and has a
bagatelle board. This room is entered from the open
verandah only, and does not, as shown on the plan, com-
municate with the room immediately adjoining. All
these day rooms, as well as the dormitories, have oak
floors, with rounded fillets as skirtings and rounded
corners to walls and ceilings. Attached to each wing
by a cross ventilated lobby is a block containing, on
the ground floor, a cloak-room and lava- ^,
tory, and on the upper floors linen- ; \
room, housemaid's closet, lavatories, bath- \ J V7] \
rooms, and water-closets. The w.c.'s for |j j | f
day use are so placed that they are ac- \
cessible either through the cloak-rooms :X
or directly from the outside. The sani-
tary fittings are on the whole well
arranged, but it would have been better
to have had cement or terrazzo floors to
the lavatories instead of lining the wood
floors with lead. Marble tops to the ?
lavatory basins would also have been iisiiJL"?-
much better in point of appearance than 1 |:
slate, and very little, if any, more costly.
We must also enter a protest against
the metal baths with their unnecessary and objection-
able wooden casings. Eire-clay enamelled baths with
a simple rim of hard wood or enamel would have been
in every way preferable.
The verandahs in front of the smaller day rooms
are admirable features, and are not only of practical ijf j n n nTf!
utility, but add much to the architectural beauty of LjU__UIJ_U^
the exterior. The dormitories are over the day rooms, S*
and are calculated at about 80 superficial and 1,000
cubic feet per bed. Each large room has two fire- H?PARTKWOOD,NSWANlIyTkenT^'
Dec. 9, 1893. THE HOSPITAL. 159
places and each smaller room one fireplace, besides
"which hot-water pipes run through all the rooms.
There are Tobin tubes in the angles of each room, but
for some unexplained reason there is no arrangement
for warming the incoming air in cold weather. This
might very easily have been done by casing in the hot-
water pipes and mating the Tobin tubes the outlet
from the casings.
Returning now to the ground floor, at each end of
the front administrative blocks is a corridor running
northwards. These two corridors lead to the dining
hall. The western corridor is also the way for both
sexes to the chapel, and the eastern corridor gives
access to the kitchen offices, and stores.
The dining hall is a large and well proportioned
room, with a handsome roof, and is lighted by windows
on the whole of the south side, and on half the north
side. It would hold probably about half as many
patients again as there is accommodation for in the
present building. On the north side is the serving-room,
where the meals are handed out through a serving
hatch. In this room is a steam-heated hot closet, and
adjoining it is a china stove. The kitchen is imme-
diately behind the serving-room. It is fitted with a.large
Tange, two gas stoves, steamers, and a steam-heated
carving table. It is well lighted, but is scarcely large
enough for the purpose. The scullery adjoins the
kitchen,and is also somewhat small, and the provision of
sinks is certainly inadequate. Beyond the kitchen are
larder, stores and the servants' hall.
Of the chapel there is not much to be said. It is,
architecturally speaking, the weakest part of the whole
building. The architecture is plain to a fault, and the
height far too small for the width ; the encaustic tile
pavement in the chancel must be positively dangerous
for lame patients, and of the tile monstrosity on the
east wall it is difficult to speak seriously. Where
every other part is so well and effectively designed it
is a thousand pities that the chapel, which should be
the culminating point of beauty, is the poorest piece of
design in the whole building.
In a small detached building to the north-east is a
laundry, fitted with hand machinery of a somewhat
inferiortype, and a disinfecting house. This latter is
fitted with a machine which unfortunately has been
proved, by a recent law suit, to be an infringement of
an existing patent, and consequently is for the present
disused pending arrangements as to royalties.
The exterior elevations of the buildings are simple
in character, but very pleasing and picturesque. The
two lower floors are faced with stock-bricks, with red
brick dressings, and the upper floors with weather
tiling; and the roofs are covered with tiles. Internally
everything is plain almost to sternness ; the large day
rooms particularly, with their uncarpeted floors and
bare walla, look terribly dreary, but we have no doubt
^eid will soon remedy this defect,
lne buildings were designed by Mr. E. B. I'Anson,
architect to St. Bartholomew's Hospital.

				

## Figures and Tables

**Figure f1:**